# Mental health among healthcare workers during COVID-19: a study to oversee the impact of the risk perception and relationship with inflammation from blood-based extracellular vesicles

**DOI:** 10.3389/fpubh.2025.1560129

**Published:** 2025-08-21

**Authors:** Coraline Canivet, Thalie Hébert, Eric Boilard, Charles M. Morin, Jessica Deslauriers

**Affiliations:** ^1^Neurosciences Axis, Centre de Recherche du Centre Hospitalier Universitaire (CRCHU) de Québec-Université Laval, Québec City, QC, Canada; ^2^Infectious and Inflammatory Diseases Axis, CRCHU de Québec-Université Laval, Québec City, QC, Canada; ^3^Department of Microbiology and Immunology, Faculty of Medicine, Université Laval, Laval, QC, Canada; ^4^CERVO Brain Research Centre, Québec City, QC, Canada; ^5^Psychology School, Université Laval, Québec City, QC, Canada; ^6^Faculty of Pharmacy, Université Laval, Québec, QC, Canada

**Keywords:** healthcare workers, preventive measures, psychological distress, insomnia, job stress, extracellular vesicles, inflammation

## Abstract

**Introduction:**

Preventive measures have been implemented in hospitals during COVID-19, but how these guidelines affected mental health among healthcare workers (HCWs) remains to be determined. On another note, reliable psychological and blood-based markers are needed to promptly identify HCWs at-risk to develop distress. Extracellular vesicles (EVs) originating from brain cross the blood–brain barrier and are detectable in blood, giving them a highly valuable potential for biomarker discovery. In HCWs with or without psychological distress, we investigated how perceived stress during COVID-19 impacted mental health. We then longitudinally evaluated the inflammatory cargo from neuron-, astrocyte-, and microglial-derived EVs that may be associated with psychological distress.

**Methods:**

Our prospective study that included an initial visit (02/2021–08/2021), and two follow-up visits 3 and 6 months later (last visit; 03/2022). HCWs (*n* = 15) completed questionnaires for perception of risk, COVID-19-specific posttraumatic symptomatology, psychological distress and burnout, as well as sleep quality. Blood was collected at each visit to characterizing inflammation from brain-derived EVs. Multiple regressions were conducted for all psychological/biological parameters based on the HCWs’ final score for psychological distress.

**Results:**

Onset of psychological distress was associated early hyperarousal. Moreover, severe distress was associated with increased astrocyte-specific levels of anti-inflammatory interleukin-10 and pro-inflammatory interferon-ɣ.

**Discussion:**

Our findings—that need to be replicated in larger studies—suggest that early hyperarousal may be predictive of later onset of psychological distress in HCWs. They also unravel a novel area of biomarker discovery study in psychiatry as inflammation from brain-derived EVs could help targeting “at-risk” individuals.

## 1 Introduction

Professional distress and burnout cost more than $20 billion to the Canadian government, and more than 30% of physicians reporting exhaustion and reduced performance at work (1).

The recent COVID-19 pandemic constituted an unprecedented situation forcing the World Health Organization to declare an international public health emergency in March 2020. In Canada, more than 4,950,000 reported infections and 68,000 deaths have been reported as of September 2024. During prior infectious outbreaks such as the severe acute respiratory syndrome (SARS), 2009 H1N1 influenza flu, and Middle East respiratory syndrome, healthcare workers (HCWs) have faced “a high risk of infection and inadequate protection from contamination, overwork, frustration, discrimination, isolation, patients with negative emotions, a lack of contact with their families, and exhaustion” (2). Meta-analyses, published during the early stages of the COVID-19 pandemic, highlighted the deleterious mental health outcomes during the COVID-19 pandemic, with a higher prevalence in HCWs vs. the general population (3, 4). Persistent fear (i.e., fear of being infected or infecting a close relative) shaped their capacity to deliver health care and were associated with poor mental health (5). Public authorities provided hospitals with guidelines (i.e., training, protective equipment) to prevent infection among their employees, but how implemented preventive measures affect fear toward risk for infection (perception and/or protection) and mental health outcomes in HCWs remain to be determined (6). While accessibility to vaccination decreased rates for posttraumatic stress disorder (PTSD), new virus variants were associated with rising incidence of PTSD (3).

However, not only the number of cases of the recent COVID-19 pandemic is far greater than the 2002–2003 SARS epidemic, but its basic reproduction number (R0) is significantly higher (7). Chinese studies found that early on 70% of frontline HCWs showed signs of distress, and 30% suffer from insomnia due to the COVID-19 outbreak (8, 9). Altogether, these factors highlight the unprecedented pressure that healthcare professionals have experienced and the need to reduce and prevent such a burden. Importantly, there is an urgent need to find psychological and accessible (i.e., blood-based) physiological markers to identify and promptly take charge of those HCWs that are at risk for prolonged disability due to distress, burnout or insomnia resulting from their work during a global health emergency crisis.

Numerous clinical studies support a role for inflammation as a pathophysiological mechanism underlying stress-related and sleep disorders (10). Professional burnout and persistent insomnia have been associated with higher circulating levels of tumor necrosis factor-*α* (TNF-α) and other pro-inflammatory factors in workers (11–13). Still, few data are available on the bidirectional relationship between inflammation, professional distress, and insomnia in HCWs, especially in the context of a global health crisis. Importantly, a limitation of the focus on whole plasma levels of inflammatory markers in biomarker discovery studies is that they do not reflect neurological changes, as their tissue and cellular origins cannot be precisely determined.

Extracellular vesicles (EVs) are secreted membrane vesicles (40–100 nm in diameter) produced by most cell types, including central nervous system (CNS) cells such as neurons, astrocytes, and microglia (14). Separating these EVs based on their cellular origin may be a major challenge. As EVs can cross the blood–brain barrier, astrocyte- (ADEs), neuron- (NDEs), and microglia-derived (MDEs) EVs can be readily detected in plasma by magnetic immunocapture with antibodies for markers specific to each brain cell type. Indeed, glutamate aspartate transporter (GLAST), L1 cell adhesion molecule (L1CAM) and transmembrane protein 119 (TMEM119) markers show specificity for astrocytes (15), neuronal (16), and microglial (17) cells, respectively. Importantly, EVs are cargo that can store and release inflammatory cytokines far from their origin (18). CNS biomarkers for brain diseases usually require invasive cerebrospinal fluid sampling as plasma levels may not be related to CNS-specific origins. As they are measurable in plasma, brain-derived EVs may be an incredible tool to develop accessible blood-based biomarkers of psychiatric disorders (19–21). Emerging evidence strengthens the reactive response of brain-derived exosomes in distinct mental health context (e.g., psychosis or major depressive disorder (MDD)), and supports their contribution to psychopathologies in proof-of-concept studies, in which brain-derived exosomes from mentally ill patients induces psychiatric-like behaviors in rodents (22–24). With inflammation known as a core mechanism in mental pathophysiologies (10), enriching EVs from specific brain cell types and studying their inflammatory content would allow us to define a specific neuroimmune biosignature associated with psychological distress-related symptomatology.

During the COVID-19 global pandemic, the HCWs faced a multifactorial context that is distinct from their usual daily lives (i.e., newly triggering and traumatizing event interacting with posttraumatic symptoms, and implemented preventive measures). In a longitudinal prospective study on HCWs from *Centre Hospitalier Universitaire* (CHU) *de Québec* healthcare facility, we aimed to: (1) determine whether individual perceptions of the implemented preventive measures in hospitals affected the psychological distress among HCWs; (2) establish the predictive relationship between posttraumatic symptoms, burnout, sleep and psychological distress; and (3) identify brain-derived inflammatory markers that are measurable in blood and associated with psychological distress in a context of the global health crisis.

## 2 Materials and methods

### 2.1 Recruitment

Male or female participants (*n* = 18; 18–59 yrs. old) were initially recruited while working full-time as a *CHU de Québec* healthcare employee (doctor, nurses, physiotherapist and respiratory therapist) with direct contact with patients during the COVID-19 crisis. Exclusion criteria included: (1) past or current positive test for COVID 19, as length of the neuroinflammatory effect of past COVID-19 infection is still not known (25); (2) current nicotine or cannabis use; (3) diabetes diagnosis; or (4) depression or burnout diagnosis within the past 6 months. Each participant was met 3 times: at the initial visit (02/2021–08/2021), as well as 3 and 6 months later (last visit in 03/2022). The 3- and 6-month time points have been selected to assess the healthy, adaptive response and maladaptive, pathological consequences, respectively (26). During the first visit, all participants provided written informed consent, and demographics information were collected via a self-report survey. At each visit, all six French version questionnaires (see section 2.2.) were self-completed (27–30). Blood was drawn into EDTA-treated tubes and centrifuged to collect plasma (1,500 rpm for 10 min; storage at −80°C until use). Blood drawn have always been performed between 8h00AM and 11h30AM. Fifteen participants (*n* = 15; 1 man and 14 women) completed all three visits. The protocol was approved by the institutional review board of the *CHU de Québec* (#2021–5,394).

### 2.2 Questionnaires

#### 2.2.1 Kessler psychological distress scale (K10)

**Figure 1:**
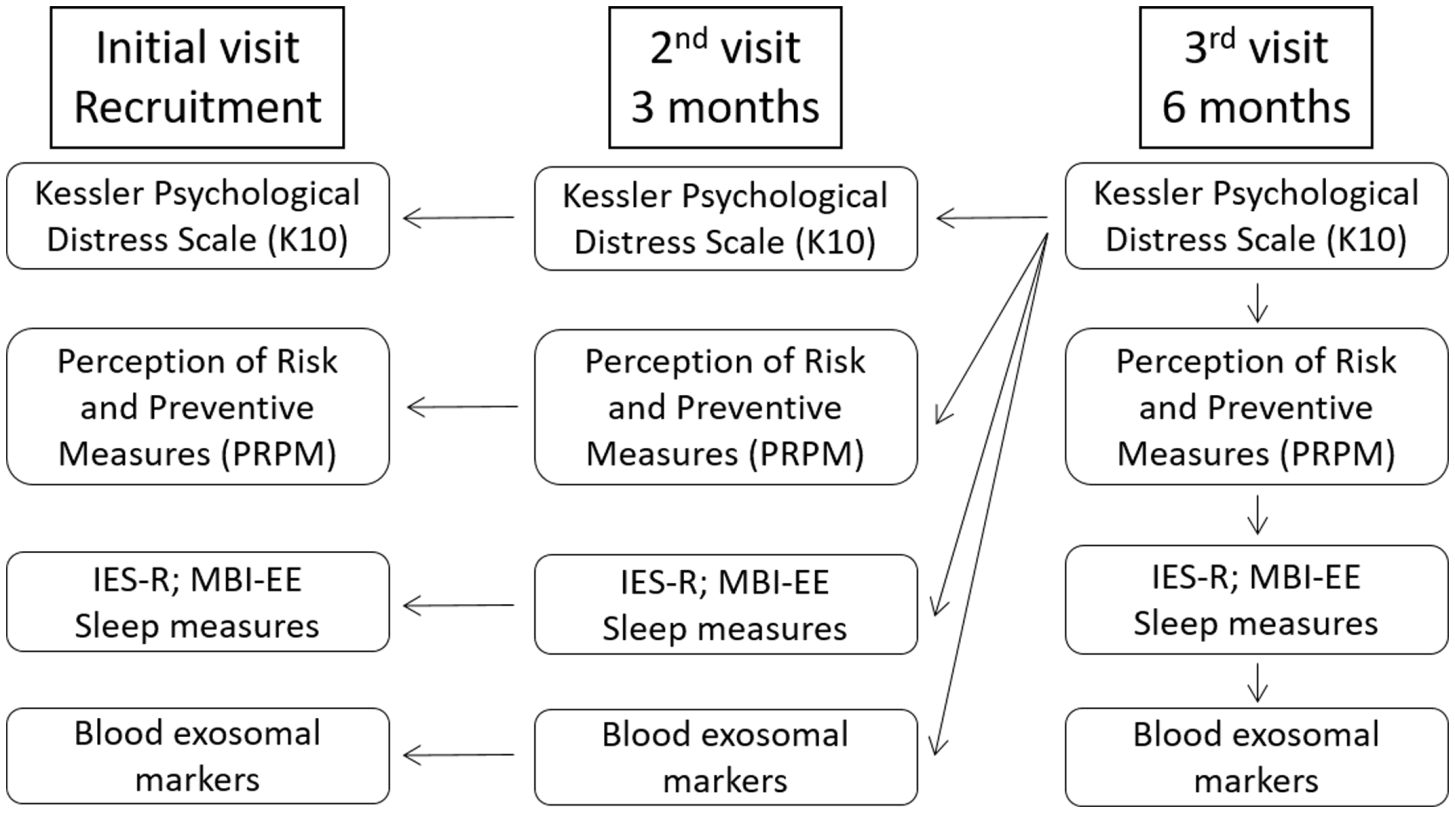
Experimental design. At the final visit, a total K10 (Kessler Psychological Distress Scale) score of 25 was used as a cutoff score to divide the participants into “no/low psychological distress” or “moderate/severe psychological distress” group. This categorization was then used as an independent variable for all other psychological and biological parameters. Insomia severity and sleep quality were assessed using the Insomnia Severity Index (ISI) and Pittsburgh Sleep Quality Index (PSQI) questionnaires, respectively. IES-R, Impact of Event Scale-Revised; MBI-EE, Maslach Burnout Inventory-Emotional Exhaustion.

The K10 survey includes 10 questions (scored from 1-none of the time to 5-all the time) to evaluate risk for psychological distress by assessing anxiety and depression symptoms experienced during the 4 preceding weeks. The psychometric properties of the K10 have been extensively examined in several civilian and occupational populations, and show very good reliability (Cronbach’s α = 0.88–0.93) (31). At the third visit, a total score of 25 (ranging from 10 to 50) was used as a cutoff score to divide the participants into “no/low psychological distress” or “moderate/severe psychological distress” group (32). This categorization was used to retrospectively compare other psychological and biological parameters (Figure 1).

#### 2.2.2 Impact of event scale revised (IES-R)

The IES-R questionnaire includes 22 questions divided into 3 subscales of symptoms: intrusion, avoidance/numbing, and hyperarousal. The IES-R used in our study has good internal consistency (α = 0.81–0.93 for its 3 subscales and total score) and reliability (correlation coefficients = 0.71–0.76 for its 3 subscales and total score) (27). Participants scored each question from 0 (not at all) to 4 (extremely) based on their COVID-19-specific experience within the 7 preceding days. The sum of all 3 scores (0–88 range) was used for statistics (27).

#### 2.2.3 Perception of risk and preventive measures (PRPM)

The PRPM questionnaire developed by Maunder and colleagues (2006) includes 18 items (scored from 1-strongly disagree to 5-strongly agree) assessing 3 distinct constructs that have satisfactory internal consistency (Cronbach’s α = 0.76–0.89): (1) adequacy of training, protection and support (i.e., training for control procedures, protective equipment, and emotional support); (2) job stress (i.e., conflict between colleagues, perceived stress, and increased workload/overtime); and (3) perception of stigma and interpersonal avoidance (i.e., coping strategies in regards to avoidance from close friends and family fearing to be contaminated). The average of all 3 scores was used for statistics (33).

#### 2.2.4 Malash burnout inventory-emotional exhaustion scale (MBI-EE)

Risk for burnout was determined using the EE subscale of the MBI questionnaire shows satisfactory internal consistency (Cronbach’s α = 0.76–0.83) while used in occupational contexts (34). The survey includes 9 items for which participant responds with a score of 0 (never) to 6 (everyday). The sum (0 to 54) was used for statistics, with a total score of ≥ 18 considered as a risk of burnout (34).

#### 2.2.5 Measures of sleep quality

Insomnia symptoms during the preceding month were evaluated with the 7-item Insomnia Severity Index (ISI) questionnaire, which has high internal consistency (Cronbach’s α = 0.88) (35). A total score (ranging from 0 to 28) of ≥ 15 considered as clinical insomnia (36). The 7-component Pittsburgh Sleep Quality Index (PSQI) is the gold standard self-administered measure of sleep, with high internal consistency (α > 0.80) across all types of populations (37). Score of each component (i.e., subjective sleep quality, sleep latency, sleep duration, sleep efficiency, sleep disturbances, use of sleep medication and daytime dysfunction) is weighted equally on a 0–3 scale. The sum of all 7 components score is used as a global PSQI score (0–21 range), for which higher score reflect poorer sleep quality (37).

### 2.3 Enrichment of astrocyte-(ADEs), microglia-(MDEs), and neuron-derived EVs (NDEs) and measurement of inflammatory content

Plasma was first treated with thrombin, then centrifuged for eliminating fibrin. Total EVs were precipitated from plasma samples using the ExoQuick Precipitation solution (System Biosciences, Inc., United States) and centrifugation (1,500 g; 1 h at 4°C) (38). With the Exo-Flow Capture kit (System Biosciences, Inc.), total EVs were incubated with magnetic-streptavidin beads coupled with biotinylated-specific antibodies: mouse anti-human biotinylated GLAST (for ADEs; Miltenyi Biotec, Inc., United States), and L1CAM (for NDEs; Invitrogen, CA) or purified anti-TMEM119 antibody (for MDEs; Biolegend, United States) that was previously biotinylated with FluoReporter™ MinioBiotin-XX labeling kit (Invitrogen, CA). Eluted GLAST-positive (ADEs), TMEM-119-positive (MDEs) and L1CAM-positive (NDEs) proteins were then quantified for several inflammatory markers (see section 2.4). Data were normalized with total protein concentrations in ADEs, MDEs, or NDEs samples as measured with the Bradford assay. Briefly, total protein concentrations were calculated as follows: [protein quantity in brain-derived EVs] = [protein quantity in total EVs] - [protein quantity in supernatant after antibody incubation]. Control quality of the enrichment of brain-derived EVs was validated via flow cytometry (Supplementary Figure 1). Levels of interferon-*γ* (IFN-γ), interleukin-1β (IL-1β), interleukin-6 (IL-6), TNF-*α*, monocyte chemoattractant protein-1 (MCP-1), interleukin-10 (IL-10), interleukin-13 (IL-13) and interleukin-1 receptor antagonist (IL-1ra) were determined in eluted ADEs, NDEs, and MDEs using multiplex V-PLEX® immunoassays (Meso Scale Discovery, United States), according to the manufacturer’s instructions.

### 2.4 Statistical analyses

For demographic parameters, tables of contingency, Fisher’s exact test (sex and profession) or Kolmogorov–Smirnov test (age) were used with GraphPad Software v9. ROUT outlier tests (Q = 0.5%) did not detect any outlier. Gaussian distribution was evaluated with Shapiro–Wilk test. All other statistical analyses were performed using the SPSS (IBM Corporation, United States). Multiple linear regressions assessed the association between the final K10 score (3^rd^ visit) and all biological (i.e., inflammatory cargo from brain-derived EVs) and psychological parameters. The time point (i.e., visit) was included as a within-subject factor, and the final K10 score was between-subject factors. The K10 score at recruitment (1^st^ visit) was included as a covariate in the regression model. A *p* value < 0.05 was considered as statistically significant. Considering *α* = 0.05 and a desired statistical power 80%, we were able to detect effect sizes (η^2^) of 0.29–0.79 for both psychological and biological variables, which are considered as large effect sizes. The design for our statistical analyses has been revised by the biostatistical services available at our research center.

## 3 Results

### 3.1 Demographic analyses

Fifteen (14 females, 93.3%; 1 male, 6.7%) of the 18 recruited participants completed all 3 visits: at the recruitment visit (February to August 2021), as well as 3 and 6 months later (last visit to March 2022). 86.7% of the participants were occupying a nursing position, whereas 13.3% of participants held another appointment in which they were providing healthcare services to patients (e.g., physical therapist). After classification based on the K10 score at the final visit, 10 participants (66.7%) had no or low psychological distress, while five individuals (33.3%) had moderate or severe distress. Table 1 shows demographic info for no/low and moderate/severe distress groups. HCWs with moderate/severe psychological risk tended to be older (45.0 ± 12.3 yrs) vs. those with lower levels of distress (36.1 ± 8.1 yrs; *p* = 0.051).

**Table 1:** Demographic info from healthcare workers at CHU de Québec.

Demographic factors	No/low psychological distress	Moderate/severe psychological distress	*p*
n	10 (66.7%)	5 (33.3%)	
Sex			0.333
Male	0 (0.0%)	1 (20.0%)	
Female	10 (100.0%)	4 (80.0%)	
Profession			0.524
Nurse	8 (80.0%)	5 (100.0%)	
Other	2 (20.0%)	0 (0.0%)	
Age (mean ± SD)	**36.1 ± 8.1**	**45.0 ± 12.3**	**0.051**

Fifteen of the eighteen recruited participants completed the study. Participants were assigned to the no/low or moderate/severe psychological distress group based on their K10 score during the last (3^rd^) visit. Fisher’s exact test (sex and profession) or Kolmogorov–Smirnov test (age) was performed for statistical analyses. Bold values represent statistically different values.

### 3.2 Psychological distress in HCWs was not associated with differences in individual perceptions of the implemented preventive measures in hospitals

We first evaluated the perceived stress in the work environment using the PRPM questionnaire’s constructs: (1) adequacy of training, protection, and support received from the healthcare facility; (2) job stress; and (3) perceived stigma and interpersonal avoidance. PRPM scores were compared across all 3 visits based on their psychological distress at the final (3^rd^) visit. All constructs did not change throughout the study (i.e., no time effect or interaction with K10 score), and were not associated with psychological distress (Supplementary Table 1).

### 3.3 Early hyperarousal symptoms, not burnout or sleep quality, were associated with the development of psychological distress in HCWs

#### 3.3.1 Posttraumautic symptomatology

With the IES-R questionnaire, we assessed whether COVID-19-specific posttraumatic symptoms are associated with and/or predict psychological distress in HCWs. No association was observed between intrusion or avoidance symptoms and the category of psychological distress (Table 2). Regression analyses revealed an overall effect of final psychological distress status on hyperarousal symptoms facing the COVID-19 pandemic (F_1,2_ = 7.82; *p* < 0.05; no time × K10 interaction; Table 2). *Post-hoc* tests confirmed that HCWs with moderate/severe psychological distress displayed higher levels of hyperarousal symptoms at the 2^nd^ (*p* < 0.01) and 3^rd^ (*p* < 0.05) visits vs. their colleagues with no/low distress (Table 2).

**Table 2:** Evaluation of COVID pandemic-specific posttraumatic symptoms in healthcare workers with no/low or moderate/severe psychological distress.

Posttraumatic symptoms	Visits	No/low distress	Moderate or severe distress	K10 3^rd^ visit	Visit	Visit * K10 3^rd^ visit
Intrusion	1	8.480 ± 1.229	11.640 ± 1.768	F_1,2_ = 3.086 *p* = 0.104 η^2^ = 0.205	F_1,2_ = 0.375 *p* = 0.691 η^2^ = 0.030	F_1,2_ = 1.049 *p* = 0.366 η^2^ = 0.080
	2	5.989 ± 1.553	11.023 ± 2.235			
	3	5.715 ± 0.977	7.770 ± 1.406			
Avoidance	1	6.744 ± 1.512	9.312 ± 2.176	F_1,2_ = 2.295 *p* = 0.156 η^2^ = 0.161	F_1,2_ = 0.116 *p* = 0.891 η2 = 0.01	F_1,2_ = 0.310 *p* = 0.736 η^2^ = 0.025
	2	3.360 ± 1.364	7.280 ± 1.963			
	3	4.245 ± 1.004	6.310 ± 1.445			
Hyperarousal	1	4.064 ± 1.057	6.671 ± 1.521	**F**_**1,2**_ **= 7.815** *p* = 0.016* η^**2**^ **= 0.394**	F_1,2_ = 0.040 *p* = 0.961 η^2^ = 0.003	F_1,2_ = 0.606 *p* = 0.554 η^2^ = 0.048
	2	**1.938 ± 0.743**	**6.125 ± 1.070** ^**‡‡**^			
	3	**2.569 ± 0.628**	**5.063 ± 0.904** ^**‡**^			
Total score	1	19.288 ± 3.062	27.624 ± 4.407	F_1,2_ = 3.807 *p* = 0.075 η^2^ = 0.241	F_1,2_ = 0.088 *p* = 0.916 η^2^ = 0.007	F_1,2_ = 0.211 *p* = 0.811 η^2^ = 0.017
	2	10.999 ± 3.382	21.002 ± 4.867			
	3	12.529 ± 2.152	19.143 ± 3.097			

Intrusion, avoidance, hyperarousal, and overall symptoms were evaluated using the Impact of Event Scale-Revised (IES-R) questionnaire. Participants were divided into no/low vs. moderate/severe psychological group based on the K10 score at the final (K10 3^rd^ visit). Data are presented as the marginalized mean ± SD. *p ≤ 0.05 for main regression effect; ‡*p* ≤ 0.05 and ‡‡*p* ≤ 0.01 following *post-hoc* comparisons with the corresponding (i.e., same visit) no/low group. Bold values represent statistically different values.

#### 3.3.2 Risk of burnout and insomnia

The final K10 score did not affect the risk for burnout (MBI-EE) or ISI-reported insomnia severity in HCWs (Supplementary Table 2), as the score remained below the cutoff score for clinical insomnia. In regard to the self-reported PSQI questionnaire, no significant difference was found on sleep quality, latency, duration and efficiency, as well as on use of sleep medication and daytime dysfunction across all three visits between both distress groups (Supplementary Table 2). An interaction between time and the final K10 score was also observed (F_1,2_ = 4.956, *p* < 0.05) on sleep disturbances. HCWs with no/low distress showing lower sleep disturbance at the 2^nd^ and final visit (*p* < 0.05 vs. the average levels at the first visit; Supplementary Table 2).

### 3.4 High levels of IFN-γ and IL-10 from astrocyte-derived EVs were predictive of late moderate to severe psychological distress

Astrocyte-, microglia- and neuron-derived EVs (Table 3; Supplementary Table 3) were isolated from plasma to characterize the inflammatory cargo from distinct CNS-specific sources. In astrocyte-derived EVs, main effects of the final K10 category were found on IFN-*γ* (F_1,2_ = 5.61, *p* < 0.05), IL-10 (F_1,2_ = 48.90, *p* < 0.001), and IL-6 (F_1,2_ = 5.91, *p* < 0.05). Astrocyte-derived EVs from HCWs with moderate/severe psychological distress at the final visit showed high levels of IFN-γ at the 2^nd^ visit (*p* < 0.01) and IL-10 at each visit (*p* < 0.01 or *p* < 0.05) as compared to HCWs with no/low distress. A tendency for higher astrocyte-levels of IL-6 was also observed in the moderate/severe group vs. the no/low group at the 2^nd^ visit (*p* = 0.066; Table 3). A time × K10 interaction was also observed (F_1,2_ = 5.15, *p* < 0.05) on TNF-*α* levels from astrocyte-derived exosomes, with HCWs with moderate/severe distress showing higher levels of TNF-α from astrocyte-derived exosomes at the 2^nd^ visit (*p* < 0.05 vs. the average levels at the final visit; Table 3).

**Table 3:** Immune cargo from brain-derived extracellular vesicles in healthcare workers with no/low or moderate/severe psychological distress.

Immune cargo	Visits	No/low distress	Moderate or Severe distress	K10 3^rd^ visit	Visit	Visit * K10 3^rd^ visit
Astrocyte-derived exosomes						
IFN-γ	1	0.051 ± 0.026	0.052 ± 0.037	**F**_**1,2**_ **= 5.612** *p* = 0.035* η^**2**^ **= 0.319**	F_1,2_ = 0.443 *p* = 0.647 η^2^ = 0.036	F_1,2_ = 2.684 *p* = 0.089 η^2^ = 0.183
	2	**0.035 ± 0.025**	**0.188 ± 0.037** ^**‡‡**^			
	3	0.027 ± 0.029	0.038 ± 0.042			
IL-10	1	**0.002 ± 0.002**	**0.016 ± 0.003** ^**‡‡**^	**F**_**1,2**_ **= 44.899** *p* = 0.001*** η^**2**^ **= 0.789**	F_1,2_ = 1.648 *p* = 0.214 η^2^ = 0.121	F_1,2_ = 0.307 *p* = 0.738 η^2^ = 0.025
	2	**0.009 ± 0.003**	**0.029 ± 0.004** ^**‡‡**^			
	3	**0.002 ± 0.003**	**0.016 ± 0.004** ^**‡**^			
IL-6	1	0.022 ± 0.009	0.030 ± 0.013	**F**_**1,2**_ **= 5.912** *p* = 0.032* η^**2**^ **= 0.330**	F_1,2_ = 0.212 *p* = 0.810 η^2^ = 0.017	F_1,2_ = 0.808 *p* = 0.457 η^2^ = 0.063
	2	0.011 ± 0.011	0.051 ± 0.016			
	3	0.010 ± 0.009	0.024 ± 0.013			
TNF-α	1	0.016 ± 0.009	0.020 ± 0.013	F_1,2_ = 2.408 *p* = 0.147 η^2^ = 0.167	F_1,2_ = 0.673 *p* = 0.519 η^2^ = 0.053	**F**_**1,2**_ **= 5.155** *p* = 0.014* η^**2**^ **= 0.300**
	2	0.015 ± 0.007	**0.069 ± 0.010** ^***ω***^			
	3	0.027 ± 0.011	**0.006 ± 0.015**			
Neuron-derived exosomes						
MCP-1	1	0.016 ± 0.011	0.051 ± 0.016	**F**_**1,2**_ **= 8.150** *p* = 0.014* η^**2**^ **= 0.404**	F_1,2_ = 0.794 *p* = 0.464 η^2^ = 0.062	F_1,2_ = 0.877 *p* = 0.429 η^2^ = 0.068
	2	0.013 ± 0.006	0.019 ± 0.008			
	3	0.011 ± 0.007	0.030 ± 0.010			
Microglia-derived exosomes						
MCP-1	1	**0.033 ± 0.013** ^**ω**^	0.019 ± 0.019	F_1,2_ = 1.705 *p* = 0.216 η^2^ = 0.124	F_1,12_ = 0.003 *p* = 0.997 η^2^ = 0	F_1,2_ = 2.709 *p* = 0.087 η^2^ = 0.184
	2	0.030 ± 0.036	0.132 ± 0.052			
	3	**0.004 ± 0.005**	0.017 ± 0.007			

Cell type-specific exosomal levels of interferon-γ (IFN-γ), interleukin-10 (IL-10), IL-6, IL-13, IL-1β, IL-6, tumor necrosis-factor-α (TNF-α), and monocyte chemoattractant protein-1 (MCP-1) were measured with Meso Scale Discovery technology in the astrocyte-, neuron-, and microglia-derived exosomes from plasma samples during each visit. The full dataset is described in Supplementary Table 2. Data are presented as the marginalized mean ± SD (pg/mg proteins). *p ≤ 0.05; ***p ≤ 0.001; ‡*p* ≤ 0.05 and ‡‡*p* ≤ 0.01 following *post-hoc* comparisons with the corresponding (i.e., same visit) no/low group; ω *p* ≤ 0.05 following *post-hoc* comparisons with the 3^rd^ dataset for the same group. Bold values represent statistically different values.

In regards to neuron-specific levels, a main effect of final K10 score was revealed on neuron-specific levels of MCP-1 (F_1,2_ = 8.15, *p* < 0.05), with highly distressed HCWs tending to display higher levels of neuron-specific MCP-1 at the initial visit (*p* = 0.098 vs. the no/low group at the same visit; Table 3). Finally, a tendency for a time × final K10 interaction was observed for microglia-derived exosomal MCP-1 (F_1,2_ = 2.71, *p* = 0.087), with levels lowering across time in HCWs with no/low distress group (*p* < 0.05 at the 3^rd^ vs. 1^st^ visit; Table 3). Astrocyte-, neuron-, or microglia-derived exosomal expression of immune markers that did not vary across time in both no/low vs. moderate/severe distress groups are presented in Supplementary Table 3.

## 4 Discussion

We longitudinally assessed the perception of HCWs toward the implemented preventive measures to limit COVID-19 infection within the healthcare facility, as well as their posttraumatic symptomatology, psychological distress, burnout and insomnia symptoms. Blood-based inflammatory cargo specific to brain-derived EVs was also characterized at several time points across a 6-month period. Participants were assigned to the no/low or moderate/severe distress group based on their final K10 psychological score, and their responses to the questionnaires were retrospectively analyzed to identify biopsychosocial markers that are associated with and/or are predictive of psychological distress.

We found that HWCs with moderate/severe distress tended to be older than their colleagues with no/low psychological distress. In line with our observation, age and years of experience of HCWs were positively associated with mean scores for perceived stress and depression during the COVID-19 pandemic (39). As older workers with extensive experience have increased workload, responsibilities and overtime, they may endorse higher levels of stress and be more at-risk for psychological distress (40). It cannot be excluded that older HCWs may also have additional responsibilities outside of work than their younger colleagues. On another note, HCWs older than 50 years were more at risk to develop severe outcomes and recovered more slowly following COVID-19 infection (41). These acknowledged risks may have increased the HCWs’ perceived stress and mental health issues. We also observed an unbalanced distribution of biological sex and occupation in the cohort, with only one male participant and 86% of participants being nurses, which is a female-predominant occupation. Biological sex has been consistently reported as a predictor of different mental health conditions, with women more at risk for major depressive disorder, anxiety, and PTSD (42, 43). In the COVID-19 context, a recent umbrella review of 87 meta-analyses examining HCWs’ mental health did not revealed sex-specific adverse mental outcomes and perceived stress (44). In line with our study, most of the meta-analyses were exclusively recruiting nurses or had higher proportions of nurses within their sample (45–48). Though Maunder and colleagues found increased risk for emotional exhaustion in nurses vs. other healthcare professionals during the COVID-19 pandemic (49), the umbrella review reported no specific effect of the job category on mental health symptoms (44).

We found that psychological distress was not associated with perceived adequacy of training, job stress, or stigma/interpersonal avoidance. In a large cohort of 1875 HCWs across 12 Ontario hospitals, increased risk due to personal protective equipment predicted adverse psychological outcomes (30). Discrepancies between this investigation and our study may be explained by our limited sample size, as well as by the highest proportion of nurses in our study. Despite the reported high efficacy of personal protective equipment and hygiene measures applied during the pandemic, nurses were at higher risk for infection and psychological impact (50). Another investigation involving 539 HCWs from 2 Ontario hospitals in Fall 2020 and Winter 2021 revealed that high self-efficacy for COVID-19 prevention and control correlated with decreased psychological distress (49). Altogether with our findings, this study suggests that risk for psychological distress may be modulated mostly by coping strategies, not perceived stress. Indeed, maladaptive coping strategies (e.g., behavioral disengagement, self-blame, and venting) were predictors of psychological distress, while humor and positive reframing were negatively associated with (51). Psychosocial factors (e.g., marriage, education, work department) and lifestyle habits (e.g., practice of mindfulness, exercise), which were not assessed in our study, may affect the perception of job stress and its management (52, 53). Still, the implementation of preventive measures and training remains critical for minimizing the harmful effects on mental health and quality of health services available to the population (54).

High psychological distress was not associated with COVID-19-specific avoidance or intrusion symptoms. Importantly, early hyperarousal symptoms were predictive of later onset psychological distress. In a longitudinal study colliding psychological data during four COVID-19 waves from 2019 to 2023 in China, exaggerated startle response and hyperarousal were the central symptoms across all four waves (55). Altogether with large meta-analyses reporting posttraumatic symptoms in 32% of HCWs during the COVID-19 pandemic (3, 56), our data suggest that hyperarousal symptoms while facing the health crisis may be a specific psychological factor that may be monitored to promote mental health surveillance among HCWs. Interestingly, fear for personal health has been shown as the strongest predictor of PTSD symptoms in HCWs during the COVID-19, underscoring the urgent need for targeted mental health interventions (57).

Recent meta-analyses reported that burnout and insomnia affected 37–42% of HCWs during the COVID-19 pandemic (3, 4). Sleep disturbances have also been associated with posttraumatic stress disorder, depression or anxiety in front-line HCWs during the COVID-19 outbreak (40, 56). Here, HCWs with high psychological distress did not report changes in sleep parameters, whereas reduced sleep disturbances were observed across time in HCWs with no/low distress. This finding may suggest that HCWs not developing high levels of distress were using resources to promote sleep quality, and facilitate resilience during the pandemic. A strength of our investigation was that both objective and subjective questionnaires were used, adding robustness to our observed associations between psychological distress and sleep quality.

Circulating (i.e., total blood levels regardless of their cellular origin) immune factors as biomarkers of psychiatric disorders face limitations with the limited specificity to neurological changes and to the heterogenous symptomatology of mental disorders (20–22). The emergence of brain-derived EVs as a novel approach to study neurological changes has led to the identification of biomarkers of neurodegeneration in rodents (58, 59), non-human primates (60), and humans (38). Here we studied temporal changes in levels of pro- and anti-inflammatory regulators released by astrocyte-, microglia-, and neuron-derived EVs from the HCWs’ blood samples. Symptoms of distress in HCWs were associated with increased astrocyte-specific levels of TNF-*α* as early as the second visit. As TNF-α is a known key player in several autoimmune diseases and regulator of CNS functional homeostasis in healthy state (61), altered levels of TNF-α from astroglial EVs may have a harmful effect in HCWs with severe psychological distress. Moreover, the onset of psychological distress in HCWs was related to early increases in astrocyte-specific levels of pro-inflammatory IL-6 and IFN-*γ*. Combat veterans with PTSD exhibit higher circulating blood levels of IFN-γ, in parallel with elevated levels of T helper lymphocytes and lower levels of regulatory T cells (62). IL-6 and IFN-γ have been associated with in depressive-like behavior, fatigue, and sleep alterations in rodents (63). We also found an early increase in levels of anti-inflammatory IL-10 in astrocyte-derived EVs from HCWs with moderate/severe psychological distress. In line with our findings, increased circulating (i.e., non-CNS-specific) levels of IL-10 have been reported in individuals with MDD or suicidal ideation (10). In addition to astrocyte-specific immune changes, HCWs reporting moderate/severe distress displayed non-detectable levels of microglia or neuron-specific mediators early on during the pandemic. In the CNS, astrocytes represent the most abundant cell type in the brain, which might explain our brain cell-specific detectable variabilities in inflammatory cargo. Overall, we demonstrated that the neuroimmune mechanisms underlying psychological distress in HWCs are specific to the astroglial cell type, and that early immune alterations from brain origins may be associated with late psychological distress. The contribution of immune content from EVs to a psychological condition is complex and a lot of work remains to be done to enhance our understanding of their roles. To our knowledge, our study is the first one evaluating the inflammatory cargo released by brain-derived EVs in association with the severity of self-reported psychological symptoms and demonstrating its feasibility. It is to be noted that the term “EVs” instead of “exosomes” were used following a general consensus from experts for “particles naturally released from the cell that are delimited by a lipid bilayer and cannot replicate” (64). Further investigations in the field of psychiatry may use these brain-derived markers to distinguishing symptoms of the large psychiatric spectrum and to creating specific biotypes.

Of note, our longitudinal investigation included limitations. First, only 15 participants (out of 90 participants as a primary objective) completed the study, limiting our statistical power and our capacity to exclude potential Type 1 or 2 statistical errors. Our statistical plan was revised accordingly with the help of the biostatistics platform from the *CHU de Québec-Université Laval* research center, and reported significantly moderate to large effect sizes on hyperarousal symptoms and immune regulators from astrocyte-derived EVs. Given the increased risk for false-positive or false-negative interpretation, caution is warranted in regards to the generalization of the findings (65), and further studies including larger sample sizes are needed.

Our limited sample size may be explained by several factors. First, the overtime work schedules that HCWs were encountering during the COVID-19 outbreak was a challenge for our recruitment, as they were required to provide our team with a blood sample in addition to completing all the questionnaires. Second, past positive test for COVID-19 was considered as an exclusion criterion to avoid confounding bias on neuroinflammation (i.e., brain-derived EVs), excluding a substantial portion of HCWs who have been exposed to the virus, and narrowing our targeted population. The selective inclusion of HCWs without any past COVID-19 positive test throughout the study may generate a selection bias, as the study cohort may not me representative of the sample population. As all HCWs had to pass three mandatory screening tests per week, latent COVID-19 infection in our cohort is unlikely. Moreover, comparing our psychological and biological parameters between non-infected and infected HCWs would have needed a much larger sample size. As history of past COVID-19 infection has been associated with increased risk for psychological symptoms (e.g., anxiety, depression, and PTSD) (66), further investigation should verify whether past COVID-19 infection interacts with the association between psychological distress and blood-based EV markers. We also observed an unbalanced distribution of biological sex and occupation in the cohort, with only one male participant and 86% of participants being nurses. Though this limited the statistical power to identify sex- and occupation-dependent outcomes, a recent umbrella review reported no specific effect of sex or job category on mental health outcomes (44). Finally, the collected data on symptomatology were self-reported, thus not associated with confirmed clinical diagnosis in order to identify blood-based biomarkers of a specific psychiatric diagnosis. Further studies should include a larger sample of participants with better distribution of sex and occupation, include non-HCWs as controls, and incorporate medical and medication (e.g., immunosuppressive) information. Importantly, studies of brain-derived EVs as blood-based biomarkers of diagnosis and risk need to be conducted in other psychiatric contexts to develop of novel avenues in the diagnosis and treatment of several mental illnesses.

## 5 Conclusion

In sum, the implementation of preventive measures in health facilities remains essential to minimize adverse effects on health and on the quality of health services available to the population, but the perceived adequacy of training, stigma and interpersonal avoidance did not impact the severity of psychological distress in our sample of HCWs. Though replication studies with larger sample sizes are needed, our study highlighted that early reported hyperarousal symptoms were associated with late psychological distress symptoms in HCWs. Lastly, late psychological distress tended to be associated with early neuroinflammatory EVs changes specific to astrocytes. All in all, our findings suggest that psychological markers (i.e., hyperarousal) and blood-based biomarkers (i.e., immune cargo from brain-derived EVs) should be investigated further for early identification of HCWs “at-risk” for psychological distress to provide them with timely and adequate support. Our study also unravels the valuable potential of brain-derived EVs for early biomarkers of risk in psychiatry.

## Data Availability

The raw data supporting the conclusions of this article will be made available by the authors, without undue reservation.
