# Supplementary figures and images for: Mental health among healthcare workers during COVID-19: a study to oversee the impact of the risk perception and relationship with inflammation from blood-based extracellular vesicles
Source: Front Public Health. 2025 Aug 21;13:1560129. doi: 10.3389/fpubh.2025.1560129 (PMC12408313; doi:10.3389/fpubh.2025.1560129)

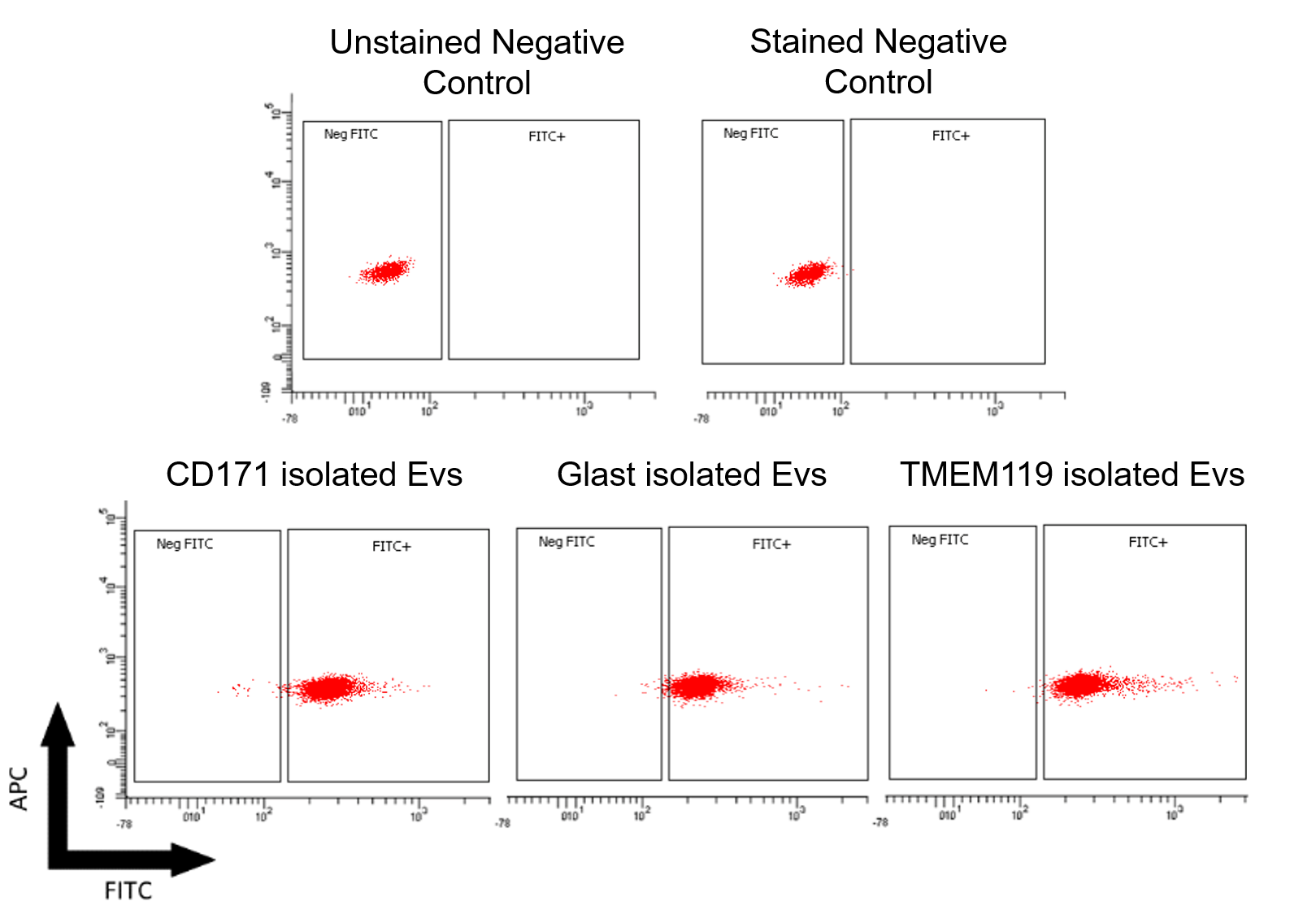

Supplement: Supplementary file 1 [file Image_1.png]
