# Supplementary material for: Mental health among healthcare workers during COVID-19: a study to oversee the impact of the risk perception and relationship with inflammation from blood-based extracellular vesicles
Source: Front Public Health. 2025 Aug 21;13:1560129. doi: 10.3389/fpubh.2025.1560129 (PMC12408313; doi:10.3389/fpubh.2025.1560129)
Supplement: Supplementary file 2 [file Table_1.docx]

**Supplemental Table 1. Evaluation perceived stress symptoms in healthcare work environment**

| **Perceived stress symptoms** | **Visits** | **No/low distress** | **Moderate or Severe distress** | **K10 3^rd^ visit** | **Visit** | **Visit * K10 3^rd^ visit** |
| --- | --- | --- | --- | --- | --- | --- |
| Adequacy of training protection and support | 1 | 2.401 ± 0.326 | 2.598 ± 0.469 | F_1,2_ = 0.156  *p* = 0.700  η^2^ = 0.013 | F_1,2_ = 2.137  *p* = 0.140  η^2^ = 0.151 | F_1,2_ = 0.609  *p* = 0.552  η^2^ = 0.048 |
|  | 2 | 2.227 ± 0.279 | 2.168 ± 0.401 |  |  |  |
|  | 3 | 2.200 ± 0.327 | 2.645 ± 0.471 |  |  |  |
| Job stress | 1 | 3.602 ± 0.400 | 4.637 ± 0.576 | F_1,2_ = 1.911  *p* = 0.192  η^2^ = 0.137 | F_1,2_ = 0.583  *p* = 0.566  η^2^ = 0.046 | F_1,2_ = 0.412  *p* = 0.667  η^2^ = 0.033 |
|  | 2 | 3.316 ± 0.333 | 3.889 ± 0.479 |  |  |  |
|  | 3 | 3.577 ± 0.336 | 4.325 ± 0.484 |  |  |  |
| Perception of stigma and interpersonal avoidance | 1 | 2.627 ± 0.270 | 3.075 ± 0.389 | F_1,2_ = 2.385  *p* = 0.148  η^2^ = 0.166 | F_1,2_ = 0.047  *p* = 0.955  η^2^ = 0.004 | F_1,2_ = 0.582  *p* = 0.566  η^2^ = 0.046 |
|  | 2 | 2.253 ± 0.257 | 2.637 ± 0.371 |  |  |  |
|  | 3 | 2.079 ± 0.236 | 2.938 ± 0.340 |  |  |  |

Perceived appropriateness of training protection and support, job stress and perception of stigma and interpersonal avoidance were evaluated with the Perception of Risk and Preventive Measures (PRPM) questionnaire. Participants were divided into no/low vs moderate/severe psychological group based on the K10 score at the final (3^rd^ visit). Data are presented as the marginalized mean ± SD.
