# Supplementary material for: Mental health among healthcare workers during COVID-19: a study to oversee the impact of the risk perception and relationship with inflammation from blood-based extracellular vesicles
Source: Front Public Health. 2025 Aug 21;13:1560129. doi: 10.3389/fpubh.2025.1560129 (PMC12408313; doi:10.3389/fpubh.2025.1560129)
Supplement: Supplementary file 3 [file Table_2.docx]

**Supplemental Table 2. Evaluation of risk of burnout and insomnia severity COVID symptoms in healthcare workers with no/low or moderate/severe psychological distress**

| **Burnout / Insomnia symptoms** | **Visits** | **No/low distress** | **Moderate or Severe distress** | **K10 3^rd^ visit** | **Visit** | **Visit * K10 3^rd^ visit** |
| --- | --- | --- | --- | --- | --- | --- |
| Risk of burnout | 1 | 16.850 ± 1.573 | 18.501 ± 2.264 | F_1,2_ = 1.736  *p* = 0.212  η^2^ = 0.126 | F_1,2_ = 0.362  *p* = 0.700  η^2^ = 0.029 | F_1,2_ = 0.471  *p* = 0.630  η^2^ = 0.038 |
|  | 2 | 15.197 ± 1.535 | 18.206 ± 2.210 |  |  |  |
|  | 3 | 14.793 ± 1.971 | 19.615 ± 2.836 |  |  |  |
| Insomnia severity | 1 | 11.230 ± 1.539 | 12.739 ± 2.215 | F_1,2_ = 0.584  *p* = 0.460  η^2^ = 0.046 | F_1,2_ = 2.235  *p* = 0.118  η^2^ = 0.163 | F_1,2_ = 0.042  *p* = 0.959  η^2^ = 0.003 |
|  | 2 | 8.503 ± 1.396 | 10.594 ± 2.009 |  |  |  |
|  | 3 | 7.927 ± 1.470 | 9.745 ± 2.116 |  |  |  |
| **PSQI constructs** | | | | | | |
| Subjective sleep quality | 1 | 1.448 ± 0.161 | 1.305 ± 0.231 | F_1,2_ = 0.007  *p* = 0.935  η^2^ = 0.001 | F_1,2_ = 0.292  *p* = 0.749  η^2^ = 0.024 | F_1,2_ = 1.494  *p* = 0.245  η^2^ = 0.111 |
|  | 2 | 1.232 ± 0.189 | 1.136 ± 0.272 |  |  |  |
|  | 3 | 1.163 ± 0.208 | 1.474 ± 0.300 |  |  |  |
| Sleep latency | 1 | 1.102 ± 0.349 | 1.596 ± 0.502 | F_1,2_ = 0.999  *p* = 0.337  η^2^ = 0.077 | F_1,2_ = 1.053  *p* = 0.365  η^2^ = 0.081 | F_1,2_ = 0.371  *p* = 0.694  η^2^ = 0.030 |
|  | 2 | 1.021 ± 0.348 | 1.358 ± 0.501 |  |  |  |
|  | 3 | 0.557 ± 0.274 | 1.286 ± 0.394 |  |  |  |
| Sleep duration | 1 | 0.363 ± 0.204 | 0.874 ± 0.294 | F_1,2_ = 0.231  *p* = 0.639  η^2^ = 0.019 | F_1,2_ = 1.904  *p* = 0.171  η^2^ = 0.137 | F_1,2_ = 1.876  *p* = 0.175  η^2^ = 0.135 |
|  | 2 | 0.819 ± 0.331 | 0.361 ± 0.477 |  |  |  |
|  | 3 | 0.272 ± 0.160 | 0.655 ± 0.231 |  |  |  |
| Sleep efficiency | 1 | 0.206 ± 0.235 | 0.788 ± 0.338 | F_1,2_ = 0.639  *p* = 0.440  η^2^ = 0.051 | F_1,2_ = 0.444  *p* = 0.647  η^2^ = 0.036 | F_1,2_ = 1.797  *p* = 0.187  η^2^ = 0.130 |
|  | 2 | 0.205 ± 0.204 | 0.390 ± 0.293 |  |  |  |
|  | 3 | 0.224 ± 0.139 | 0.152 ± 0.201 |  |  |  |
| Sleep disturbance | 1 | **1.954 ± 0.169** | 1.493 ± 0.244 | F_1,2_ = 0.024  *p* = 0.878  η^2^ = 0.002 | F_1,2_ = 0.962  *p* = 0.396  η^2^ = 0.074 | **F_1,2_ = 4.956**  ***p* = 0.016***  **η^2^ = 0.292** |
|  | 2 | **1.407 ± 0.166 ^ω^** | 1.786 ± 0.239 |  |  |  |
|  | 3 | **1.334 ± 0.161 ^ω^** | 1.533 ± 0.231 |  |  |  |
| Use of sleep medication | 1 | 1.238 ± 0.372 | 1.125 ± 0.535 | F_1,2_ = 0.129  *p* = 0.726  η^2^ = 0.011 | F_1,2_ = 0.878  *p* = 0.429  η^2^ = 0.068 | F_1,2_ = 1.219  *p* = 0.313  η^2^ = 0.092 |
|  | 2 | 0.995 ± 0.390 | 1.410 ± 0.562 |  |  |  |
|  | 3 | 1.071 ± 0.370 | 1.458 ± 0.532 |  |  |  |
| Daytime dysfunction | 1 | 1.478 ± 0.262 | 1.244 ± 0.377 | F_1,2_ = 0.767  *p* = 0.398  η^2^ = 0.060 | F_1,2_ = 0.062  *p* = 0.940  η^2^ = 0.005 | F_1,2_ = 2.039  *p* = 0.152  η^2^ = 0.145 |
|  | 2 | 1.157 ± 0.249 | 1.485 ± 0.359 |  |  |  |
|  | 3 | 0.750 ± 0.200 | 1.500 ± 0.288 |  |  |  |
| Global PSQI score | 1 | 7.788 ± 1.082 | 8.424 ± 1.558 | F_1,2_ = 0.651  *p* = 0.435  η^2^ = 0.051 | F_1,2_ = 1.268  *p* = 0.300  η^2^ = 0.096 | F_1,2_ = 1.970  *p* = 0.161  η^2^ = 0.141 |
|  | 2 | 6.837 ± 1.145 | 7.926 ± 1.648 |  |  |  |
|  | 3 | 5.370 ± 0.994 | 8.059 ± 1.430 |  |  |  |

Risk of burnout and insomnia severity were evaluated with the “Malash Burnout Inventory-EE” and “Insomnia Severity Index” questionnaires, respectively. Seven constructs of the Pittsburgh Sleep Quality Index (PSQI) self-report questionnaire were also evaluated. Data are presented as the marginalized mean ± SD. * *p* ≤ 0.05 for main regression effect; ^ω^ *p*  ≤ 0.05 following post hoc comparisons with the 1^st^ dataset for the same group.
