# Supplementary material for: Mental health among healthcare workers during COVID-19: a study to oversee the impact of the risk perception and relationship with inflammation from blood-based extracellular vesicles
Source: Front Public Health. 2025 Aug 21;13:1560129. doi: 10.3389/fpubh.2025.1560129 (PMC12408313; doi:10.3389/fpubh.2025.1560129)
Supplement: Supplementary file 4 [file Table_3.docx]

**Supplemental Table 3. Immune cargo from brain-derived extracellular vesicles in healthcare workers with no/low or moderate/severe psychological distress**

| **Immune  cargo** | **Visits** | **No/low distress** | **Moderate or Severe distress** | **K10 3^rd^ visit** | **Visit** | **Visit * K10 3^rd^ visit** |
| --- | --- | --- | --- | --- | --- | --- |
| **Astrocyte-derived exosomes** | | | | | | |
| IL-13 | 1 | 0.420 ± 0.126 | 0.396 ± 0.182 | F_1,2_ = 0.434  *p* = 0.522  η^2^ = 0.035 | F_1,2_ = 0.632  *p* = 0.540  η^2^ = 0.050 | F_1,2_ = 0.174  *p* = 0.842  η^2^ = 0.014 |
|  | 2 | 0.363 ± 0.185 | 0.551 ± 0.267 |  |  |  |
|  | 3 | 0.200 ± 0.133 | 0.347 ± 0.191 |  |  |  |
| IL-1β | 1 | 0.098 ± 0.043 | 0.041 ± 0.062 | F_1,2_ = 1.223  *p* = 0.290  η^2^ = 0.093 | F_1,2_ = 0.669  *p* = 0.522  η^2^ = 0.053 | F_1,2_ = 2.497  *p* = 0.103  η^2^ = 0.172 |
|  | 2 | 0.030 ± 0.021 | 0.143 ± 0.030 |  |  |  |
|  | 3 | 0.020 ± 0.017 | 0.059 ± 0.025 |  |  |  |
| IL-1ra | 1 | 0.819 ± 0.367 | 0.447 ± 0.528 | F_1,2_ = 0.198  *p* = 0.664  η^2^ = 0.016 | F_1,2_ = 0.088  *p* = 0.916  η^2^ = 0.007 | F_1,2_ = 0.873  *p* = 0.431  η^2^ = 0.068 |
|  | 2 | 1.591 ± 0.691 | 1.272 ± 0.995 |  |  |  |
|  | 3 | 1.110 ± 1.546 | 3.404 ± 2.225 |  |  |  |
| MCP-1 | 1 | 0.024 ± 0.012 | 0.040 ± 0.018 | F_1,2_ = 0.005  *p* = 0.945  η^2^ = 0.000 | F_1,2_ = 0.021  *p* = 0.979  η^2^ = 0.002 | F_1,2_ = 0.548  *p* = 0.585  η^2^ = 0.044 |
|  | 2 | 0.021 ± 0.010 | 0.014 ± 0.014 |  |  |  |
|  | 3 | 0.016 ± 0.009 | 0.005 ± 0.013 |  |  |  |
| **Neuron-derived exosomes** | | | | | | |
| IFN-𝛾 | 1 | 0.067 ± 0.023 | 0.034 ± 0.033 | F_1,2_ = 0.330  *p* = 0.576  η^2^ = 0.027 | F_1,2_ = 0.165  *p* = 0.849  η^2^ = 0.014 | F_1,12_ = 0.058  *p* = 0.943  η^2^ = 0.005 |
|  | 2 | 0.043 ± 0.022 | 0.020 ± 0.032 |  |  |  |
|  | 3 | 0.083 ± 0.052 | 0.081 ± 0.074 |  |  |  |
| IL-10 | 1 | 0.018 ± 0.003 | 0.008 ± 0.005 | F_1,2_ = 0.864  *p* = 0.371  η^2^ = 0.067 | F_1,2_ = 0.204  *p* = 0.817  η^2^ = 0.017 | F_1,2_ = 0.195  *p* = 0.824  η^2^ = 0.016 |
|  | 2 | 0.014 ± 0.006 | 0.013 ± 0.009 |  |  |  |
|  | 3 | 0.032 ± 0.013 | 0.018 ± 0.019 |  |  |  |
| IL-13 | 1 | 0.421 ± 0.112 | 0.233 ± 0.162 | F_1,2_ = 1.477  *p* = 0.248  η^2^ = 0.110 | F_1,2_ = 0.365  *p* = 0.698  η^2^ = 0.029 | F_1,2_ = 1.355  *p* = 0.277  η^2^ = 0.101 |
|  | 2 | 0.303 ± 0.137 | 0.431 ± 0.197 |  |  |  |
|  | 3 | 0.921 ± 0.292 | 0.234 ± 0.420 |  |  |  |
| IL-1β | 1 | 0.094 ± 0.019 | 0.060 ± 0.027 | F_1,2_ = 1.278  *p* = 0.280  η^2^ = 0.096 | F_1,2_ = 0.280  *p* = 0.758  η^2^ = 0.023 | F_1,2_ = 0.983  *p* = 0.389  η^2^ = 0.076 |
|  | 2 | 0.042 ± 0.022 | 0.056 ± 0.032 |  |  |  |
|  | 3 | 0.216 ± 0.083 | 0.056 ± 0.120 |  |  |  |
| IL-6 | 1 | 0.040 ± 0.007 | 0.009 ± 0.010 | F_1,2_ = 0.814  *p* = 0.385  η^2^ = 0.064 | F_1,2_ = 0.457  *p* = 0.639  η^2^ = 0.037 | F_1,2_ = 0.704  *p* = 0.488  η^2^ = 0.058 |
|  | 2 | 0.009 ± 0.009 | 0.020 ± 0.012 |  |  |  |
|  | 3 | 0.043 ± 0.019 | 0.033 ± 0.027 |  |  |  |
| TNF-α | 1 | 0.041 ± 0.008 | 0.020 ± 0.011 | F_1,2_ = 0.765  *p* = 0.399  η^2^ = 0.060 | F_1,2_ = 1.437  *p* = 0.257  η^2^ = 0.107 | F_1,2_ = 0.532  *p* = 0.594  η^2^ = 0.042 |
|  | 2 | 0.017 ± 0.012 | 0.023 ± 0.017 |  |  |  |
|  | 3 | 0.065 ± 0.029 | 0.025 ± 0.042 |  |  |  |
| IL-1ra | 1 | 1.619 ± 1.124 | 1.903 ± 1.618 | F_1,2_ = 0.980  *p* = 0.342  η^2^ = 0.075 | F_1,2_ = 0.611  *p* = 0.551  η^2^ = 0.048 | F_1,2_ = 0.345  *p* = 0.712  η^2^ = 0.028 |
|  | 2 | 1.743 ± 0.955 | -0.090 ± 1.374 |  |  |  |
|  | 3 | 1.116 ± 0.763 | 0.093 ± 1.098 |  |  |  |
| **Microglia-derived exosomes** | | | | | | |
| IFN-𝛾 | 1 | 0.106 ± 0.066 | 0.142 ± 0.095 | F_1,2_ = 0.021  *p* = 0.886  η^2^ = 0.002 | F_1,2_ = 0.526  *p* = 0.598  η^2^ = 0.042 | F_1,2_ = 0.407  *p* = 0.670  η^2^ = 0.033 |
|  | 2 | 0.130 ± 0.035 | 0.038 ± 0.051 |  |  |  |
|  | 3 | 0.002 ± 0.006 | 0.013 ± 0.009 |  |  |  |
| IL-10 | 1 | 0.014 ± 0.006 | 0.014 ± 0.009 | F_1,2_ = 0.083  *p* = 0.778  η^2^ = 0.007 | F_1,2_ = 0.467  *p* = 0.632  η^2^ = 0.037 | F_1,2_ = 0.149  *p* = 0.862  η^2^ = 0.012 |
|  | 2 | 0.015 ± 0.007 | 0.014 ± 0.010 |  |  |  |
|  | 3 | 0.010 ± 0.004 | 0.003 ± 0.006 |  |  |  |
| IL-13 | 1 | 0.442 ± 0.222 | 0.823 ± 0.319 | F_1,2_ = 0.014  *p* = 0.908  η^2^ = 0.001 | F_1,2_ = 0.290  *p* = 0.751  η^2^ = 0.024 | F_1,2_ = 1.899  *p* = 0.172  η^2^ = 0.137 |
|  | 2 | 0.850 ± 0.250 | 0.437 ± 0.360 |  |  |  |
|  | 3 | 0.147 ± 0.073 | 0.271 ± 0.104 |  |  |  |
| IL-1β | 1 | 0.058 ± 0.031 | 0.113 ± 0.044 | F_1,2_ = 1.033  *p* = 0.329  η^2^ = 0.079 | F_1,2_ = 0.408  *p* = 0.669  η^2^ = 0.033 | F_1,2_ = 2.151  *p* = 0.138  η^2^ = 0.152 |
|  | 2 | 0.089 ± 0.022 | 0.007 ± 0.032 |  |  |  |
|  | 3 | 0.022 ± 0.116 | 0.292 ± 0.167 |  |  |  |
| IL-6 | 1 | 0.019 ± 0.017 | 0.060 ± 0.024 | F_1,2_ = 1.573  *p* = 0.234  η^2^ = 0.116 | F_1,2_ = 1.122  *p* = 0.342  η^2^ = 0.086 | F_1,2_ = 1.612  *p* = 0.220  η^2^ = 0.118 |
|  | 2 | 0.036 ± 0.011 | 0.024 ± 0.015 |  |  |  |
|  | 3 | 0.000 ± 0.003 | 0.014 ± 0.004 |  |  |  |
| TNF-α | 1 | 0.019 ± 0.014 | 0.051 ± 0.020 | F_1,2_ = 0.311  *p* = 0.587  η^2^ = 0.025 | F_1,2_ = 0.576  *p* = 0.570  η^2^ = 0.046 | F_1,2_ = 1.701  *p* = 0.204  η^2^ = 0.124 |
|  | 2 | 0.051 ± 0.015 | 0.030 ± 0.021 |  |  |  |
|  | 3 | 0.011 ± 0.007 | 0.023 ± 0.011 |  |  |  |
| IL-1ra | 1 | 0.459 ± 0.229 | 0.237 ± 0.330 | F_1,2_ = 0.649  *p* = 0.436  η^2^ = 0.051 | F_1,2_ = 0.064  *p* = 0.938  η^2^ = 0.005 | F_1,2_ = 1.706  *p* = 0.203  η^2^ = 0.124 |
|  | 2 | 0.404 ± 0.370 | 0.295 ± 0.533 |  |  |  |
|  | 3 | -0.037 ± 0.150 | 0.093 ± 0.216 |  |  |  |

Cell type-specific exosomal levels of interferon-γ (IFN-γ), interleukin-10 (IL-10), IL-6, IL-13, IL-1β, IL-6, tumor necrosis-factor-α (TNF-α), and monocyte chemoattractant protein-1 (MCP-1) were measured with Meso Scale Discovery technology in the astrocyte-, neuron-, and microglia-derived exosomes from plasma samples during each visit. Data are presented as the marginalized mean ± SD (pg/mg proteins).
